# Polyvinyl alcohol film comprising biochar modified titanium dioxide nanocomposites as decoloring and disinfectant agents

**DOI:** 10.1038/s41598-025-87432-7

**Published:** 2025-04-03

**Authors:** Ahmed F. Ghanem, Abdelrahman A. Badawy, Ahmed A. Youssef, Naema S. Yehia, Farag A. Issa, Manal A. Nofal

**Affiliations:** 1 Packaging Materials Department, Chemical Industries Research Institute, National Research Centre, 33 El Behooth St., Dokki, Giza, Egypt; 2 Physical Chemistry Department, Advanced Materials Technology and Mineral Resources Research Institute, National Research Centre, Giza, 12622 Egypt; 3 Chemistry Department, Faculty of Science, Menofiya University, Shebein El- Kom, Egypt

**Keywords:** Titanium dioxide, Biochar, Nanocomposites, Water treatment, Disinfection, Nanoparticles, Organic-inorganic nanostructures

## Abstract

In this work, titanium dioxide nanowires were prepared hydrothermally in strong alkaline medium. In parallel, nanostructural biochar was obtained via carbonization of rice husk at relatively high temperature. Then, titanate nanowires were modified with the nanorods of biochar via in-situ and ex-situ approaches in order to determine the best way to produce the nanocomposites with improved properties. Polyvinyl alcohol was used as a commercial matrix to include the superlative nanocomposite obtained and casted as a free-standing nanocomposite film. The synthesized nanowires, nanorods, and their nanocomposites were intensively investigated with transmission electron microscope (TEM), scanning electron microscope (SEM), energy dispersive X-ray (EDX), Fourier transform infrared (FTIR), X-ray diffraction (XRD), and N_2_ gas sorption. The microscopic images confirmed successful preparation and modification of nanostructures. FTIR showed strong interactions between the surface functional groups of the obtained nanomaterials. XRD exhibited a reduction in the crystallite size upon the treatment step. Also, surface texture analysis of titanate nanowires displayed a significant enhancement, particularly in terms of surface area and total pore volume. These superior properties promote the obtained nanocomposites to be evaluated in the water treatment compared with the pristine. The results confirmed complete removal of methylene blue (20 ppm) from the synthetic wastewater within only 20 min. in dark either by using the nanocomposites as powders or even as films. Kinetics and isotherms indicated that the adsorption process obeyed Langmuir model and follows pseudo-second order. On the other hand, the prepared materials depicted a strong biocidal activity against pathogenic microorganisms. The obtained nanocomposites may open opportunities towards developed adsorbents with superior features and performance for applications in the field of water decontamination.

## Introduction

There are several uses for dyes, including printing, dyeing, paper, painting, and textiles^1^. However, because they are poisonous, nonbiodegradable, and carcinogenic substances, dyes are among the most perilous types of water pollution^2^. Various approaches have been employed to eliminate colors from the effluents. Examples include ozonation, photocatalysis, evaporation, reverse osmosis, ion exchange, filtering, solvent extraction, flotation, and electrochemical oxidation^3–7^. Moreover, biological approaches are widely utilized for decolorizing and detoxifying wastewater. These methods mostly involve aerobic, anaerobic, or their combination^8,9^. However, in terms of the cost, the energy consumption, the treated water quality, and the process complexity, most of these techniques have plentiful shortcomings^10–12^. Adsorption is currently one of the most promising mode of treatment because of its ease of use, high capacity, high efficiency, and good regeneration ability^13,14^. Abundant numbers of materials, namely known as adsorbents, have been synthesized to eliminate cationic and anionic dyes from wastewater^14–17^.

Among the well-known adsorbents, titanium dioxide (TiO_2_) has paid great attention in water treatment applications thanks to its excellent physicochemical properties and low cost^18^. TiO_2_ can be fabricated in different nanostructures including nanoparticles, nanotubes, flower-like, and nanorods^19–22^. Particularly, titanate nanowires (TNWs) are one-dimensional nanomaterial with unusual lattice structure and properties such as large surface area, unique surface chemistry, and tunable transport features^23^. TNWs can be obtained utilizing several methods such as electrochemical, thermal oxidation, and hydrothermal^24^. The latter approach involves boiling titanium dioxide nanoparticles in an alkaline medium under high pressure and temperature in order to grow the nanoparticles into nanowires according to the previously reported mechanism^25^. This method is simple, inexpensive, and produces ultralong and nano-width wires with a high yield. In the field of water treatment, titanium dioxide nanostructure is generally combined with the wastewater in the dark or even under illumination forming a suspension^26^. TiO_2_ can demineralize the wastewater with a high efficiency especially in light via photocatalysis^27^. Besides, titanium dioxide can act as an adsorbent for a wide range of pollutants including heavy metals, dyes, and microorganisms. In our previous work, TNWs showed excellent performance in lead ions removal^28^. However, using nano titanium dioxide as fine powders sometimes causes particle aggregation and decreases surface reactive sites^29^. Furthermore, additional filtration processes are a must to separate the suspended particles^30^. Moreover, its surface area is not comparable to other adsorbents including carbon-based materials. Therefore, in order to overcome the drawbacks of restricted active sites and avoid particle aggregation along with an easy operational system, TiO_2_ can be modified and incorporated in a polymer matrix. For instance, hybrid cellulose membranes modified with TNWs coated with iron oxide and copper oxide nanoparticles as filters for bacteria and virus^31^. Another intensive review presented the recent tends of cellulose included with TiO_2_ nanocomposite and their potential applications in water treatment^32^. A very interesting study by Azeroual et al. showed high adsorption of methylene blue and safranin dyes using cryogel beads contain sodium alginate/titanium dioxide nanowire doped with zirconium^33^.

As commonly known, carbonaceous materials have found strong workability as adsorbents thanks to their intriguing properties. Particularly, biochar (BC) is a solid carbon byproduct obtained during the pyrolysis of carbon-rich biomass at relatively low temperatures^34^. The physicochemical properties of the produced biochar rely on the source of combusted material^35^. As a cost-effective adsorbent, BC has unique characteristics in terms of large surface area, plenty of surface functional groups, high porosity, and high adsorption capacity^36^. Therefore, biochar has emerged as a promising solution to various environmental challenges, in particular, water decontamination^36^. Several reports have been released discussing the efficiency of different sourced biochar as adsorbents for dyes^37–40^. Also, biochar can be obtained in the nanoscale and different morphologies such as nanoparticles and nanorods^41–43^. To improve their properties, people have utilized biochar as a filler for polymer matrices to be eventually employed in the removal of pollutants from the wastewater^43–48^. Incorporation of the nanomaterials in the polymer matrix and formulations as films, gel beads, or fibers could overcome the accompanied challenges of using powder nanomaterials and enhance the efficiency of whole nanocomposite^46,49,50^. In our previous work, biochar nanorods improved the performance of polyacrylamide hydrogel against the removal of phenol^43^.

According to the previous discussion and as a trial to overcome the shortcomings of titanate nanowires as an adsorbent by exploiting their unique features, biochar nanorods were used to modify the surface of titanium dioxide nanowires. In this work, TNWs were prepared via hydrothermal method using TiO_2_ nanoparticles as a precursor in alkaline medium. Then, the obtained sodium titanate nanowires were modified via in-situ and ex-situ approaches with biochar nanorods that produced from carbonization of rice husk. The modified nanowires were incorporated with polyvinyl alcohol matrix so that free-standing nanocomposite films were achieved. The prepared samples were investigated with TEM, SEM, FTIR, XRD, and gas sorption. Also, the characterized samples were evaluated against both of methylene blue removal and microbial growth. Kinetics and adsorption reaction orders were also studied.

## Materials and methods

### Materials

Titanium dioxide nanoparticles (P25) and sodium hydroxide pellets were purchased from Degussa (USA). Polyvinyl alcohol (PVA, medium molecular weight) was provided from Alfa Aesar (Germany). Rice husk was supplied by Egyptian farmers in Menofiya Governorate. Before using, the collected samples were rinsed well with deionized water. Then, the washed husks were dried in the vacuum oven at ~ 100 °C overnight.

### Synthesis of titanium dioxide nanowires (TNWs)

**Fig. 1:**
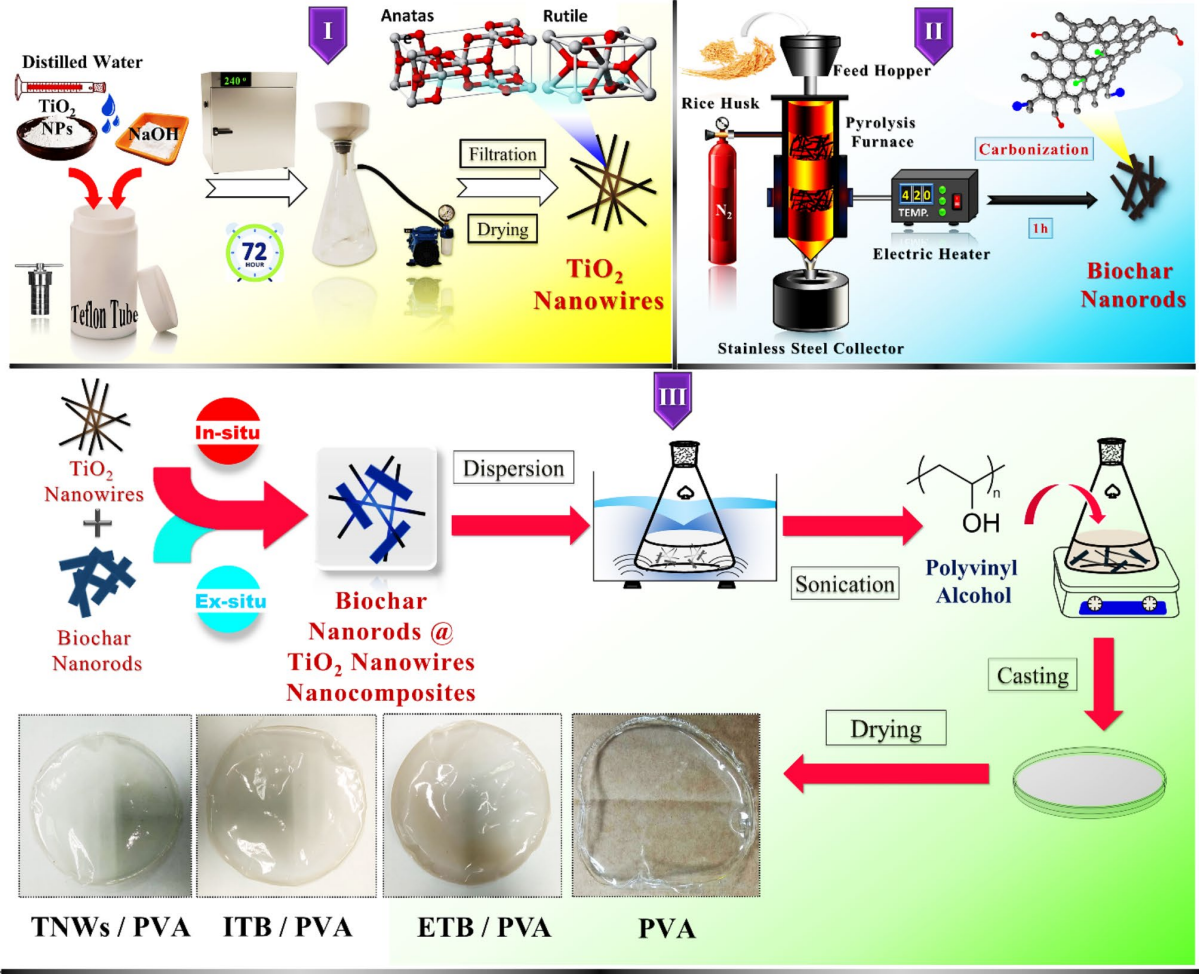
Experimental representation for synthesis of [**I**] Titanium dioxide nanowires, [**II**] Biochar nanorods, [**III**] Modified titanium dioxide nanowires and their corresponding PVA free standing nanocomposite films.

A white suspension was obtained by dispersion of 2 g of titanium dioxide nanoparticles in sodium hydroxide solution according to our earlier work^28^. Then, the mixture was heated at 240 °C in the well-sealed autoclave. After 72 h., the mixture was filtered out and washed several times with (1.0 M) sodium chloride followed by distilled water. The obtained white precipitate of titanate nanowires was collected and dried overnight at 80 ^o^C under vacuum (Fig. 1).

### Synthesis of biochar (BC)

The purified rice husk was charged into an electric furnace and pyrolyzed at 420 °C under nitrogen atmosphere for 60 min^43^. The sample color was changed into black indicating formation of biochar. After cooling, the sample was collected and kept in a stainless-steel container for further experiment (Fig. 1).

### In-situ preparation of titanium dioxide/biochar nanocomposite (ITB)

In this experiment, 10 *wt.* % of the obtained biochar was added to a dispersed solution of titanium dioxide nanoparticles (2 g) and sodium hydroxide. Then, the mixture was sonicated for 60 min. and left under vigorous stirring overnight in order to acquire a homogenous solution. After that, the grey suspension was transferred into a Teflon tube fixed in a sealed autoclave and placed in an oven at 240 °C for 72 h. under an ambient atmosphere. Once cooling, the autoclave was opened and the ingredients were transferred to a filtration system under suction. The sample was separated and well-washed by distilled water until the pH of filtrate reached normal. Finally, the resulting precipitate of modified titanate nanowires was collected and dried overnight at 80 °C under vacuum (Fig. 1).

### Ex-situ preparation of titanium dioxide / biochar nanocomposite (ETB)

The prepared titanium dioxide nanowires were modified with the obtained biochar after successfully synthesizing each individually. Typically, one gram of TNWs was dispersed in 20 mL distilled water for 30 min. Then, a fixed weight of BC (10 *wt.* %) was added and sonicated for a further 30 min. After that, the mixture was transferred to a magnetic stirrer adjusted at 300 rpm and room temperature. The milky suspension was left for 72 h. in order to achieve strong interaction through the physical mixing. Finally, the resulting mixture was centrifuged, well-washed, and dried in vacuum oven overnight at 80 °C (Fig. 1).

### Synthesis of polyvinyl alcohol nanocomposite films (PVATB)

Polyvinyl alcohol was used as a carrier for the prepared titanium dioxide nanowires and its biochar nanocomposites. Three PVA nanocomposite films were prepared, individually, by dispersing 4 *wt.* % of TNWs, ITB, and ETB, according to polymer weight, in three bottles, each contains 20 mL distilled water in a water bath sonicator for 30 min. Then, each suspension was left under vigorous stirring for 48 h. so that the highest level of dispersion was achieved. A certain weight of PVA (0.5 g) was added to each mixture and heated up 90 °C for 30 min. to dissolve the polymer pellets. Glycerol (50 µL) was also added as a plasticizer. After that, the casting solutions were kept under strong stirring for 24 h. In three glass petri dishes (10 cm), each solution was casted carefully on stand. Next, the dishes were transferred to the oven at 80 °C overnight. Finally, in order to obtain free-standing PVA nanocomposite matrices, each film was peeled off from the petri dish and kept in a sealed package put in a dissector for further usage and to avoid moisture exposure. The codes donated for the obtained films were TNWs@PVA, ITB@PVA, and ETB@PVA assigned to PVA films containing unmodified, in-situ modified, and ex-situ modified titanium dioxide nanowires, respectively. Pure PVA film was also prepared according to the previous protocol in the absence of fillers for comparison (Fig. 1).

### Techniques

The chemical structure of the prepared titanium dioxide nanowires and its nanocomposites were identified by Fourier transform infrared spectroscopy (FT-IR, Perkin Elmer).

The alteration in the crystal structure before and after modification with biochar was investigated by X-ray powder diffraction (Bruker D 8 advance target). In this analysis, The Cu Kα was used as a radiation source with a secondly monochromator (λ = 1.5405 Å) at 40 kV and 40 mA. The scanning rate was 0.2^o^ min^− 1^ for phase identification and line broadening profile analysis, respectively. The change in crystallite size was determined using the Scherrer equation,

1 $${\text{d}} = {\text{K}}\lambda /\beta _{{1/2}} \cos \theta$$

where, d is the mean crystalline diameter, λ is the X-ray wavelength, K is the Scherrer constant (0.89), β_1/2_ is the full width at half maximum (FWHM) of the main diffraction peak of the main crystalline phase, and θ is the diffraction angle.

The surface morphology of the prepared titanium dioxide and its nanocomposites along with PVA films was examined using a transmission electron microscope (TEM, JEOL JEM-1230 with acceleration voltage of about 80 kV) and scanning electron microscope (SEM, JEOL-SEM), respectively. Energy dispersive X-ray (EDX) is a hyphenated unit to SEM instrument was used for elemental analysis. From TEM micrographs and using Image J software, size distribution in terms of wires or rods width was determined and the average values were estimated from histograms. Particularly, an optical microscope (Olympus CX-23 LED binocular research microscope) was also utilized to observe the prepared pristine nanowires at low magnification.

The surface textures were studied for the prepared titanium dioxide nanowires and their biochar nanocomposites with Quantochrome Nova-Touch 4LX automated gas-sorption apparatus (USA). In which, each sample was degassed at 150 °C for 6 h. followed by N_2_ gas input at 77 K for the adsorption process. The desorption was then carried out after achieving the highest applied gas by decreasing it. The adsorption isotherms were obtained and the surface area (S_BET_) was calculated according to Brunauer–Emmett–Teller (BET) equation. Moreover, the pore volume and the pore size distribution were estimated from the desorption isotherms according to Barrett, Joyner and Halenda (BJH) method.

The biocidal potential for all prepared samples was investigated against Gram-positive bacteria (*Staphylococcus aureus*), Gram-negative bacteria (*Escherichia coli*), and yeast (*Candida albican*). In the typical procedure with some modifications to the previous report^51^, certain weight of sample (mg/mL) was incubated under shaking with (50 µL) of bacterial suspension, 0.5 McFarland standards (1.5 × 10^8^ CFU/mL), inoculated to 5 mL of specific medium for each organism in a sterilized test tube at 37 °C for 24 h. The samples were exposed to UV irradiation. The antimicrobial activity of each sample was determined by measuring the optical density (OD) on UV–vis spectrophotometer at a fixed wavelength (600 nm) according to the following equation:

2 $${\text{Reduction}}\;{\text{Growth}}\% = \left( {{\text{OD}}_{{({\text{control}})}} {-}{\text{OD}}_{{({\text{sample}})}} /{\text{OD}}_{{({\text{control}})}} } \right)*100$$

In order to illustrate the ability of the obtained PVA nanocomposite films to remove dyes from synthetic wastewater, a stock of methylene blue dye (MB) of 20 ppm initial concentration was prepared. Utilizing the adsorption mode of treatment, each nanocomposite film compared with pure one was immersed in 30 mL taken from MB’ stock in a 150 mL glass bottle with a plastic cap. Then, the mixtures were stirred at 150 rpm and room temperature in the dark. A sample was withdrawn every 20 min. over 80 min. and the remaining dye was determined by the spectrophotometer (UV-vis JASCO V-730). The removal percentage was finally calculated according to the next equation:

3 $${\text{Removal}}\% = \left( {{\text{C}}_{{\text{o}}} {-}{\text{C}}_{{\text{s}}} /{\text{C}}_{{\text{o}}} } \right)*100$$

where, C_o_ is the initial dye concentration and C_s_ is the dye concentration after treatment with the sample at a certain contact time (min.).

## Results and discussions

### Transmission electron microscope (TEM)

**Fig. 2:**
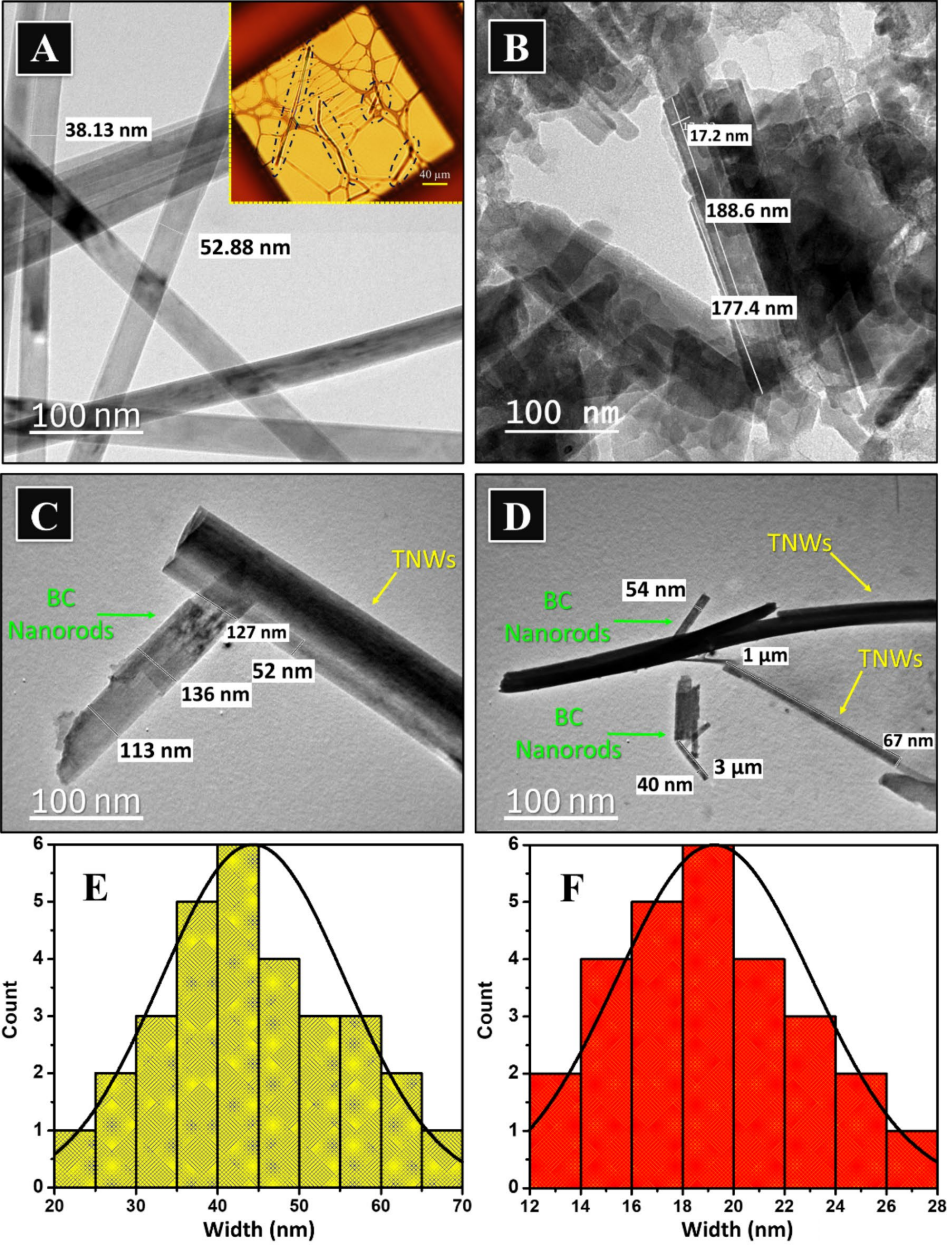
TEM micrographs for [**A**] Titanium dioxide nanowires (Inset image displays an image under optical microscope), [**B**] Biochar nanorods, [**C**] In-situ modified titanium dioxide nanowires (ITB), [**D**] Ex-situ modified titanium dioxide nanowires (ETB), and [**E**-**F**] Width distribution histograms for pristine titanium dioxide nanowires and biochar nanorods, respectively.

TEM micrographs of TiO_2_ and biochar obtained are shown in Fig. 2. The images showed that the titanium dioxide was formed as rigid nanowires in large numbers with no significant nanoparticle morphology, Fig. 2[A]. This confirmed the successful growth of TiO_2_ nanoparticles into thin ultra-long nanowires via hydrothermal method. The same observation was previously recorded^52^.The width of the produced nanowires ranges from 20 to 70 nm. However, the average wire width was determined from the histogram and found equal to 45 nm, Fig. 2[E]. On the other hand, TEM of biochar obtained via carbonization of rice husk exhibited nanorods morphology with some aggregations of irregular shapes which might be attributed to the incomplete conversion of the nanorods, Fig. 2[B]. The corresponding histogram of the obtained nanorods showed average rod width equal ~ 19 nm, Fig. 1[F]. Particularly, micrographs, depicted in Fig. 2[C-D], emphasized the successful modification of titanate nanowires by both suggested approaches, in-situ and ex-situ. The integrity of the nanowires and the nanorods was maintained during the treatment process. Moreover, the biochar nanorods seem interpentrated inside the wires or between their bundles. This might refer to the possible strong interaction between the nanorods and the nanowires. interpenetrated.

### Scanning electron microscope (SEM)

**Fig. 3:**
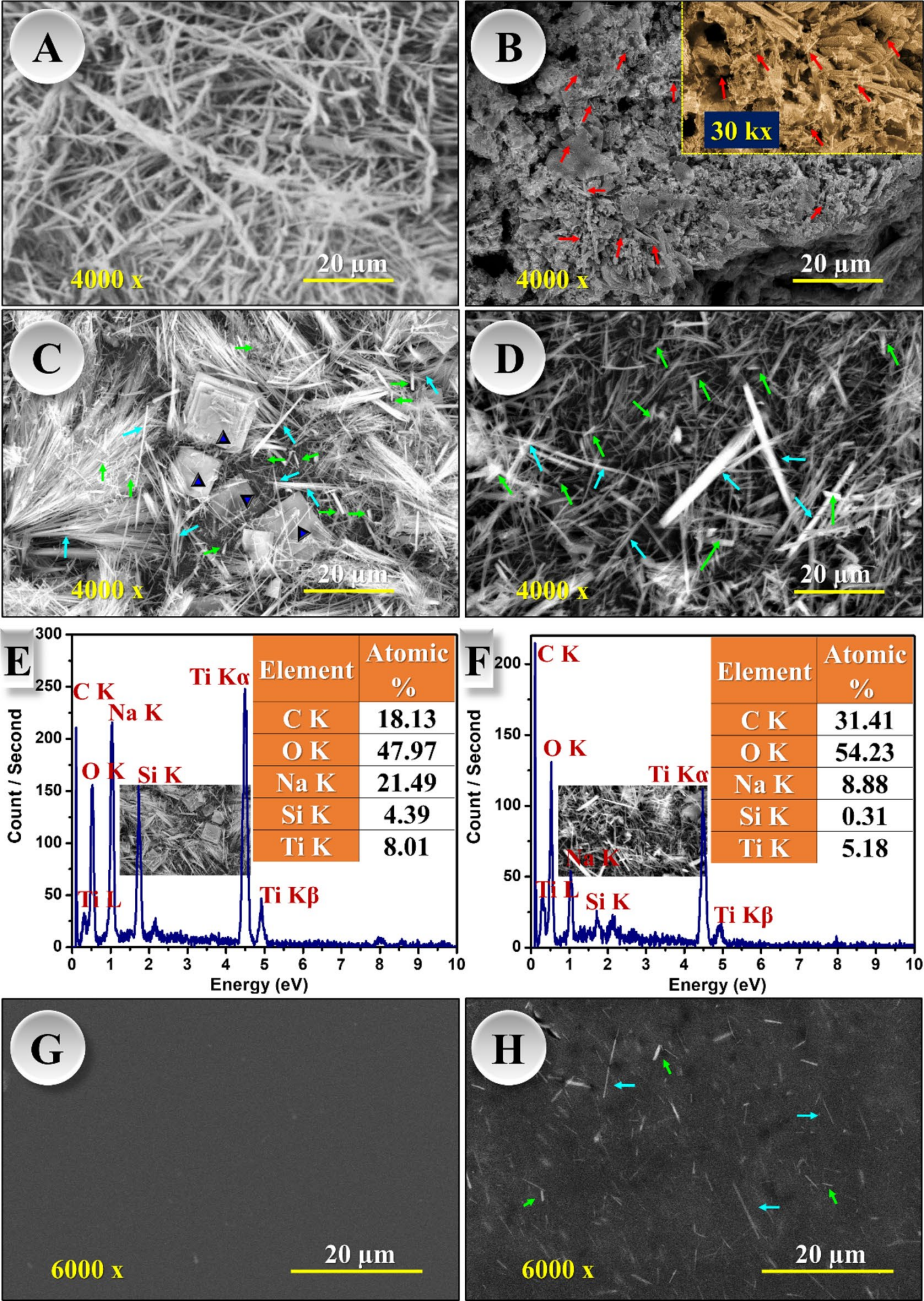
SEM images for [**A**] Titanium dioxide nanowires, [**B**] Biochar nanorods, [**C**] In-situ modified titanium dioxide nanowires (ITB), [**D**] ex-situ modified titanium dioxide nanowires (ETB), [**E**-**F**] Their EDX spectra, respectively, [**G**] Top surface of pure PVA film, and [**H**] Top surface of nanocomposite PVA film (ITB@PVA).

Figure 3 depicts the images taken by the scanning electron microscope for titanium dioxide nanowires and their modification. The titanium dioxide micrograph showed bundles of wires in the nanoscale and entangled, Fig. 3[A]. Biochar exhibited aggregates of nanorods (red arrows) can be seen at higher magnification. Additionally, some masses are observed attributed to the formed char, Fig. 3[B]. Upon modification of nanowires, the nanorods (green arrows) were seen interfered and homogenously distributed with titanium nanowires (cyan colour), Fig. 3[C-D]. However, an odd morphology of squared platelets or in other words cubic-like shape (blue triangles) appeared after in-situ modification of TWNs which might be referred to nano silicalite products. This morphology is absent in those modified via ex-situ approach. As known, biochar contains a high amount of silica^53^ which could be grown into this structure at high pH and under hydrothermal conditions. Several reports discussed the hydrothermal growth of crystalline silica from rice husk^54–56^. These results can be also confirmed by EDX, Fig. 2[E-F], as one can observe the concentration of silicon and sodium elements in the in-situ sample, Fig. 3[E], unlike in the case of ex-situ sample, Fig. 3[F] as a result of arrangement of elements in crystals at high temperature and pH. PVA has a smooth surface without significant cracks confirming the successful casting process, Fig. 3[G]. The inclusion of ex-situ modified titanium dioxide nanowires (ETB) showed well-dispersion and homogeneous distribution for both wires and rods within the matrix emphasizing good interaction between the composition Fig. 3[H].

### Fourier transform infrared (FTIR)

**Fig. 4:**
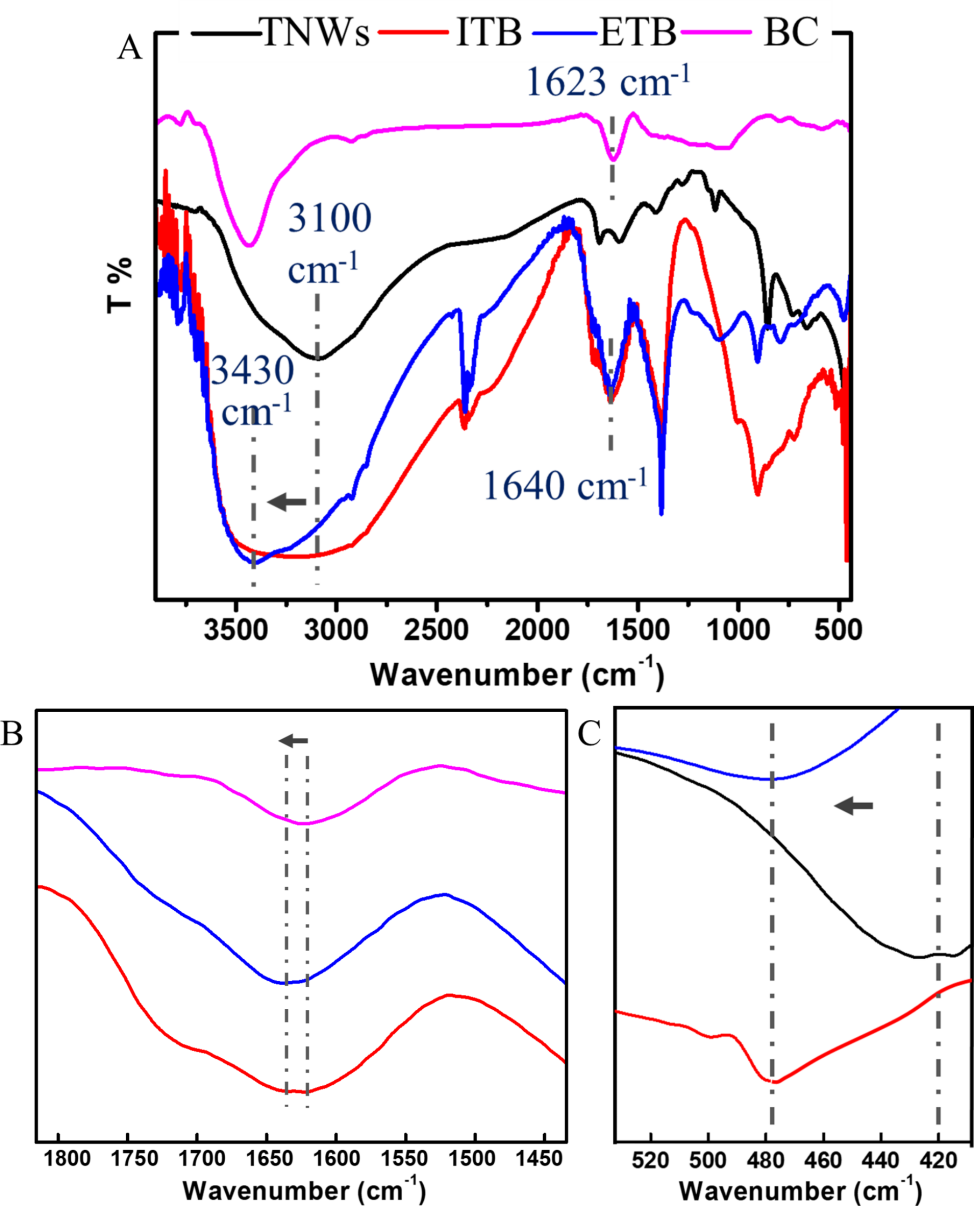
[**A**] FTIR spectra for Titanium dioxide nanowires (TNWs), Biochar nanorods (BC), In-situ modified titanium dioxide nanowires (ITB), and ex-situ modified titanium dioxide nanowires (ETB). [**B**-**C**] Zooming in FTIR peaks showing more details in the ranges from ~ 1450 to ~ 1800 cm^− 1^ and from ~ 410 to 520 cm^− 1^.

FTIR was used to investigate the mode of interaction between TNWs and BC, Fig. 4. However, the spectrum of titanium dioxide nanowires showed a broad band at ~ 3100 cm^− 1^ revealed to surface hydroxyl groups. Other bands were observed at ~ 838, ~ 517, and ~ 420 cm^− 1^ assigned to the bending and stretching vibration of Ti–O–Ti bond. These values are close to those previously reported^57,58^. Meanwhile, biochar displayed characteristic peaks at ~ 3430 and 1625 cm^− 1^ corresponding to the stretching vibration of -OH groups and C = C, respectively, in agreement with the state of the art^43^. A low-intensity band appeared at ~ 1730 cm^− 1^ attributed to the carbonyl group. Indeed, these oxygenated functional groups improved the interaction with the nanowires and the PVA matrix.

**Fig. 5:**
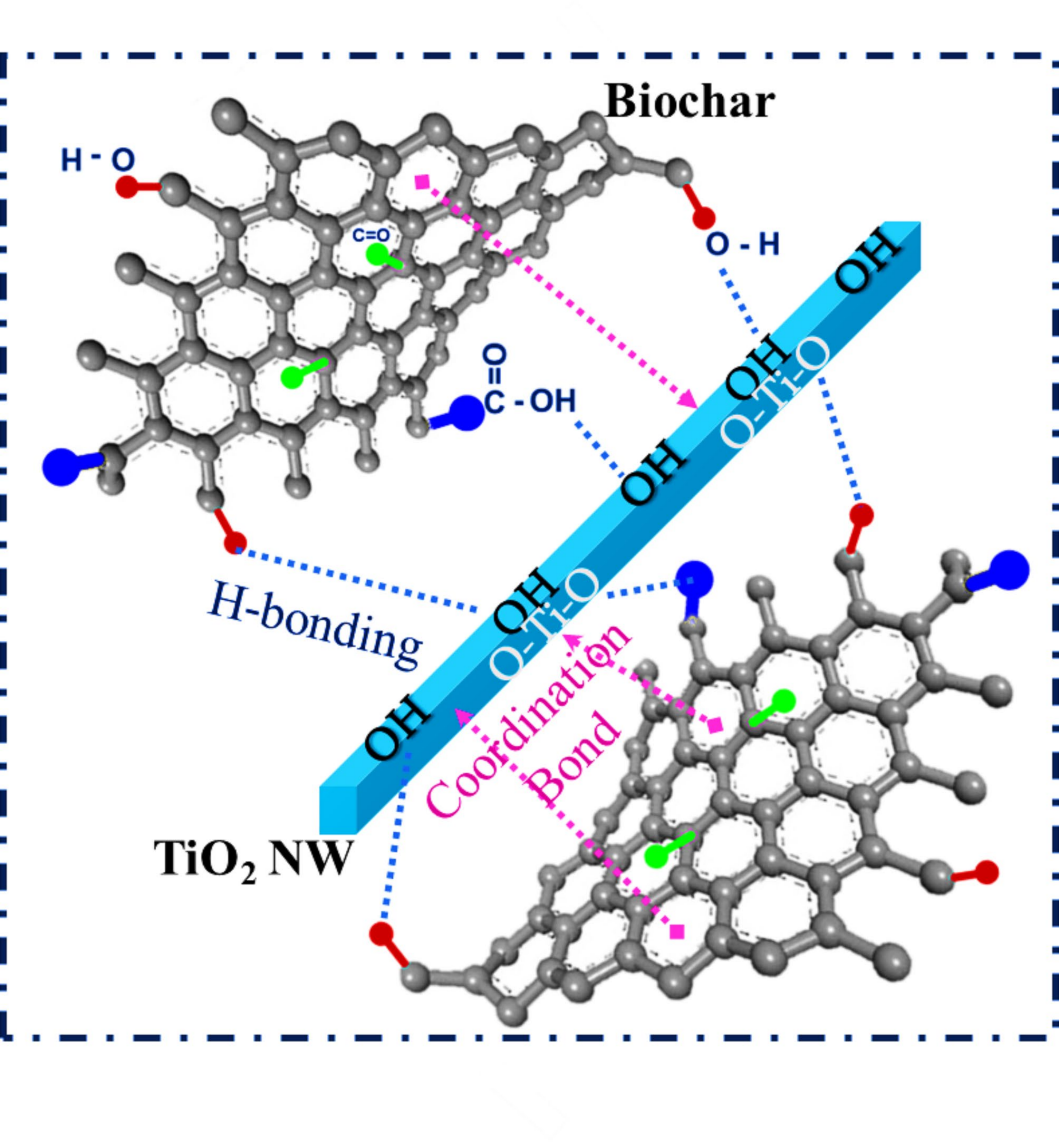
Representation for the mode of interactions between titanate nanowires and biochar nanorods.

Upon modification of titanium dioxide with biochar either by in-situ or by ex-situ, the spectra were changed. The characteristic band of TNWs at 3100 cm^− 1^ shifted to a higher wavenumber with an increase in the broadness. This might be attributed to the possible interaction through hydrogen bonding between the oxygenated functional groups at both surfaces as schematically (Fig. 5). Moreover, other shifts were observed from 1623 to 1640 cm^− 1^ and from 410 to ~ 480 cm^− 1^, Fig. 4[B-C], which might be revealed the probable coordination bond between titanium and π-cloud of the phenyl ring. Ghanem et al. confirmed the same behaviour while modification of ZnO with aromatic hyperbranched polymer^59^. FTIR confirmed the successful interaction between titanium dioxide nanowires and biochar nanorods by both methods of modification.

### X-ray diffraction (XRD)

**Fig. 6:**
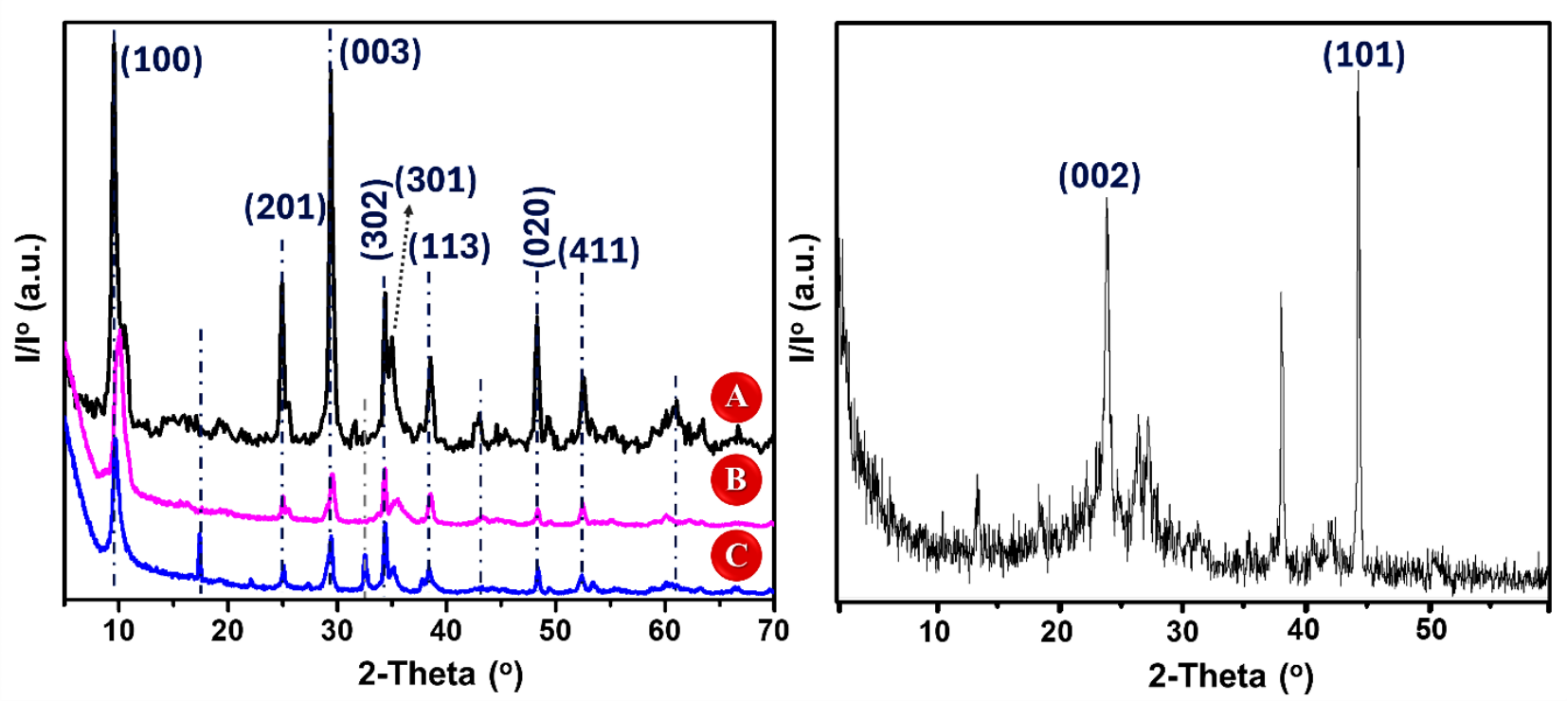
XRD spectra for [Left** A**-**C**] Titanium dioxide nanowires (TNWs), ex-situ modified titanium dioxide nanowires (ETB), and in-situ modified titanium dioxide nanowires (ITB), respectively. [Right] Biochar nanorods (BC).

In order to confirm the interaction between the nanowires and nanorods, X-diffraction was performed, Fig. 6. Particularly, TNWs showed main distinctive θ peaks of sodium titanate (Na_2_Ti_3_O_7_) phase, Fig. 6[Left A], according to (PDF No. 00-059-0666), which is also known as sodium metatitanate in agreement with the published spectrum^60,61^. Particularly, lines pronounced at 9.5°, 24.9°, 29.4°, 34.3°, 35.0°, 38.4°, 48.3°, and 52.4° reveal the Miller indices of (100), (201), (003), (302), (301), (113), (020), and (411), respectively. BC showed special lines at ~ 25° and ~ 45° assigned to (002) and (101), respectively, related to the graphitic structure confirming the decomposition of cellulosic composition into char, Fig. 6[Right]. Some other peaks can be seen at different theta values related to the metallic residues. These findings are harmonious with previous reports^62–64^. In-situ modification of nanowires, Fig. 6[Left C], exhibited a slight shift in the main peaks with an appearance of new lines at ~ 17.5° and ~ 33° which might be attributed to the phase of sodium silicate crystal, that observed in SEM, in agreement with state of art^65^. Meanwhile, ex-situ treatment, Fig. 6[Left B], showed more shift for the highest peaks, particularly, that pronounced at ~ 9.5°. This peak moved to a higher value by ~ 0.5°. The other peaks displayed a slight shift. Also, no new peaks were observed in this nanocomposite spectrum which confirmed the potential growth of silicate crystals under hydrothermal conditions at high pH supporting the results of in-situ spectrum and its SEM micrographs. In the same context, the crystallite size was measured for all samples using Sherrer equation^66^. The obtained values were found equal to 32.84, 23.46, and 18.24 nm for TNWs, ITB, and ETB, respectively. These findings emphasized that the ex-situ treatment provides the lowest crystallite size. Generally, in-situ and ex-situ modification approaches could improve the particle morphology and retard the grain growth of titanate nanowires as stated by Ghanem et al.^58^. Careful inspection in the XRD spectra, one can observe the absence of biochar main lines in the modified samples which might be attributed to the lower concentration of biochar in the tested sample in addition to the probable interactions in the nanocomposite. The same observation was previously reported in several articles^67–69^.

**Table 1:** Textural properties titanium dioxide nanowires (TNWs), in-situ modified titanium dioxide nanowires (ITB), ex-situ modified titanium dioxide nanowires (ETB)

Parameters	TNWs	ITB	ETB
S_BET_, (m^2^/g)	31.46	116.05	179.491
Correlation coefficient, (R^2^)	0.9912	0.9881	0.9985
Surface area “BJH adsorption” (m^2^/g)	13.62	120.57	165.785
Pore volume “BJH adsorption” (cm^3^/g)	0.0296	0.2741	0.37691
Pore radius “BJH adsorption” (nm)	2.405	1.6282	1.6282
Total pore volume, V_P_, (cm^3^/g)	0.03022	0.2813	0.3869
Average pore size (nm)	2.4435	4.8492	4.3109
Average particle radius (nm)	12.7823	4.1942	20.6513

### Surface area

**Fig. 7:**
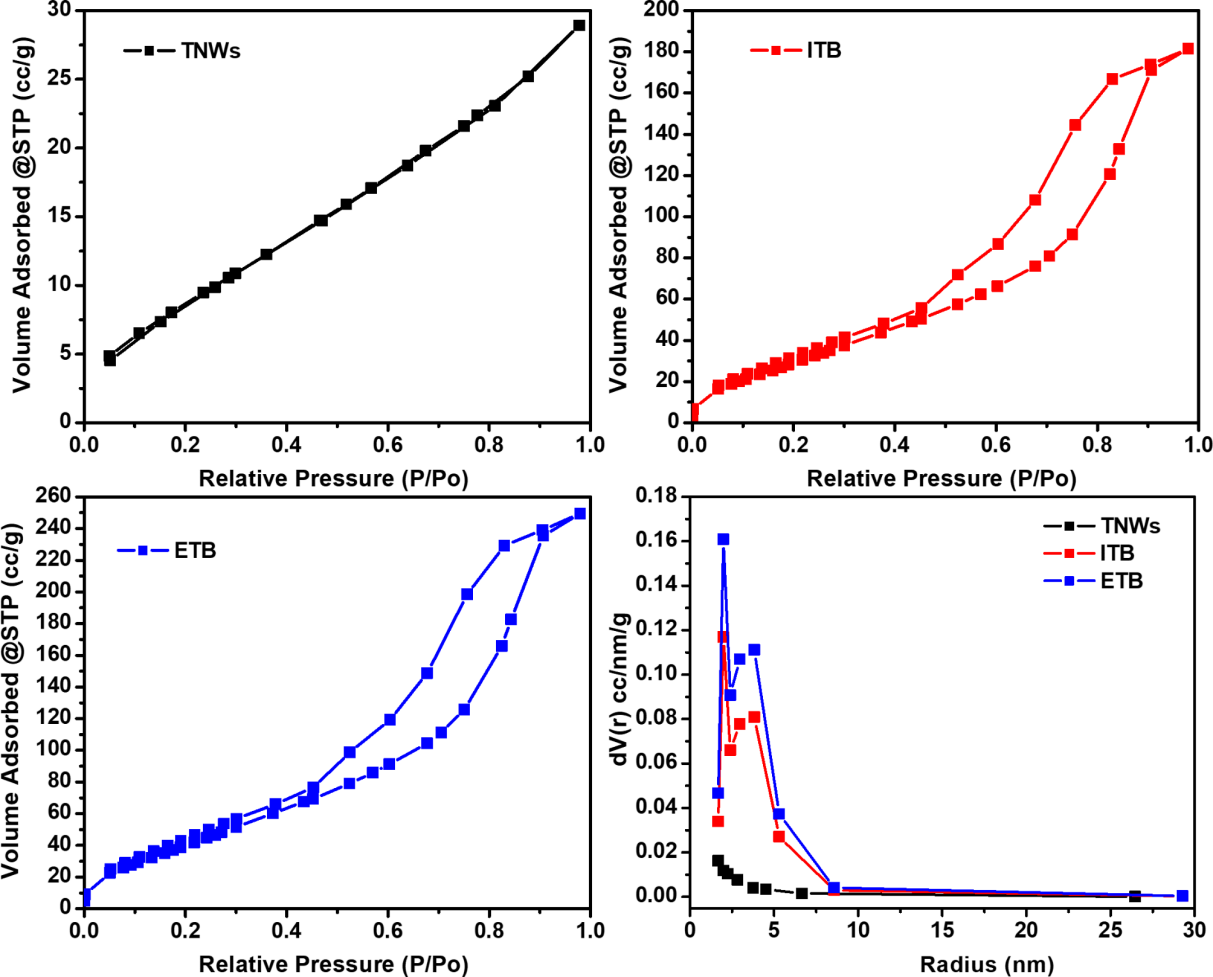
N_2_ adsorption–desorption isotherms for titanium dioxide nanowires (TNWs), in-situ modified titanium dioxide nanowires (ITB), ex-situ modified titanium dioxide nanowires (ETB), and their corresponding pore size distribution by BJH- method.

The texture profile of titanate nanowires and their modification was studied with N_2_ gas adsorption. The obtained isotherms and the pore size distribution are depicted in Fig. 7. As shown, TNWs displayed a uniform adsorption/desorption isotherm of type III without significant hysteresis and with limited gas adsorption. The same observation was recorded previously^70^. Upon the inclusion of nanorods, the surface texture was significantly altered. The amount of adsorbed gas was increased 6 times and 8 times via insertion of biochar nanorods by in-situ (ITB) and ex-situ (ETB) approaches, respectively. However, both isotherms exhibited.

Type IV of H3 type hysteresis loop in the range *P/Po* ranges from 0.3 to 0.9 due to capillary condensation by filling up and emptying of mesopore. These results emphasized the presence of a porous structure with increased porosity. Barret–Joyner–Halenda (BJH) method was used to calculate the pore size distribution of the prepared samples as depicted in Fig. 7. All samples presented a mesoporous structure i.e. the pore diameter is the range between 2 and 50 nm. However, the modified samples showed an average pore diameter of 2–5 nm. According to the acquired results, the specific surface area and the other texture parameters were calculated and summarized in Table 1. The calculated surface area of titanate nanowires was increased almost 4- and 6-fold after treatment with the nanorods via in-situ (ITB) and ex-situ (ETB) approaches, respectively. The other texture parameters, including total pore volume and average pore size, were also improved for the same reasons.

### Dye adsorption

**Fig. 8:**
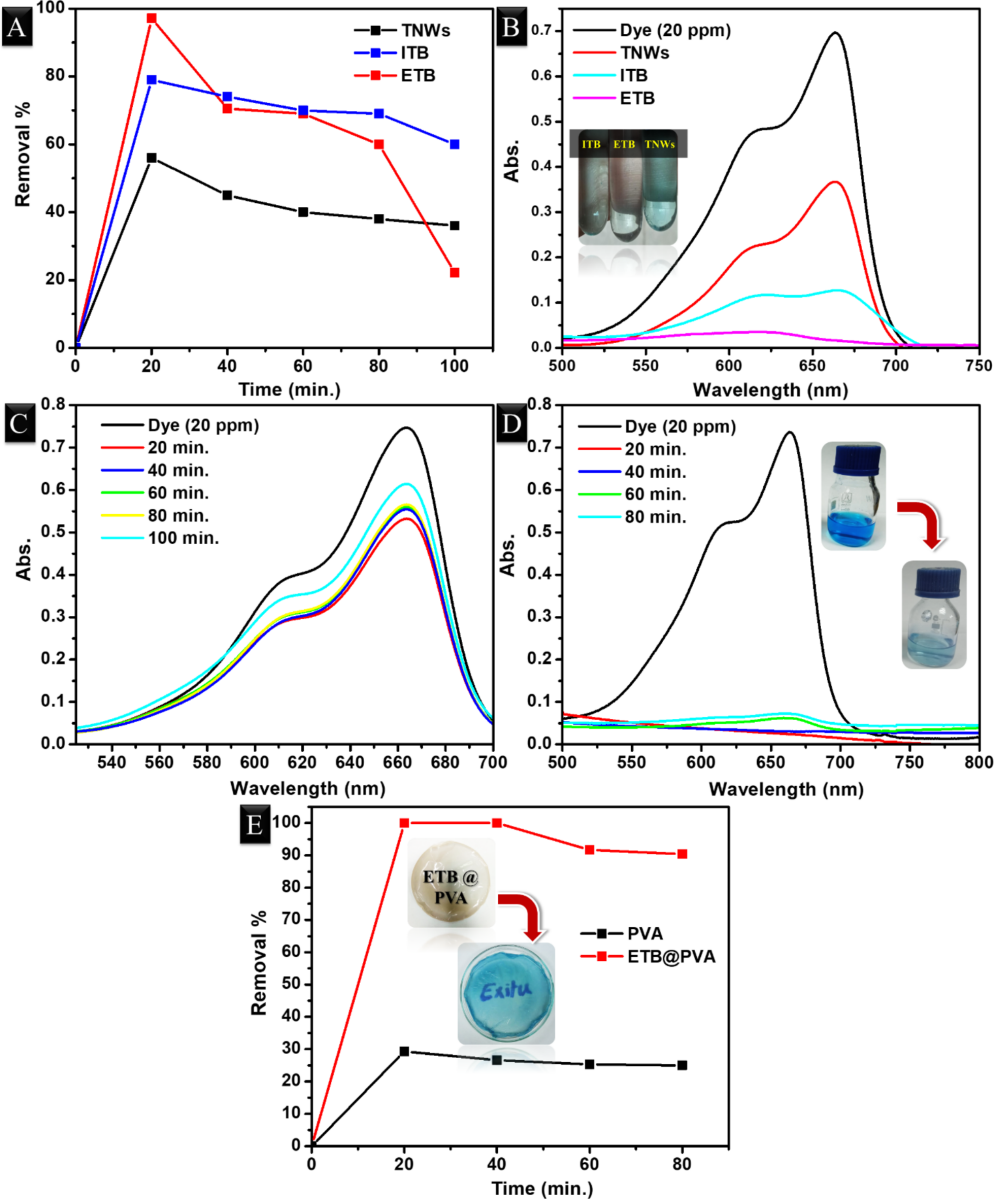
[**A**] Methylene blue removal % of modified TiO_2_ nanowires compared with pristine against contact time, [**B**] Their corresponding dye absorbance after 20 min., [**C**-**D**] Dye absorbance after adsorption over pure PVA and its composite film, respectively, (inset images are the treated water and the nanocomposite film after adsorption at the end of reaction), and [E] Their removal % against contact time.

The prepared titanium dioxide nanowires and their modification products were evaluated in the removal of methylene blue from synthetic wastewater of 20 ppm concentration under normal conditions. It is commonly known that TiO_2_ is among the strong photocatalysts as it has the ability to decompose organic pollutants under light irradiation^71^. However, in the present study, titanium dioxide was used as an adsorbent for dye in the dark. As shown in Fig. 8[A], the prepared titanate nanowires have limited adsorption of MB as it recorded MB removal of ~ 60%. Upon modification with biochar nanorods either by in-situ or by ex-situ, the removal % increased up to ~ 80% and ~ 100%, respectively. The characteristic MB peak was mostly disappeared after treatment with ETB adsorbent, Fig. 8[B]. The reason behind this improvement might be attributed to the significant increment in the surface area and the reduction in the crystallite size (cf. “X-ray diffraction (XRD)” section and “Surface area” section)^72^ i.e. ETB sample showed the highest surface area and lowest crystallite size which in turn enhanced the adsorption of dye with a high rate at the beginning of the reaction. Moreover, it is obvious thatall samples tend to make desorption after 20 min. as the removal % started to decrease by prolonged time i.e. the optimum contact time was only 20 min., Fig. 8[A]. Therefore, ETB sample was selected to be blended with the PVA matrix in order to eventually obtain nanocomposite free-standing film. This film was evaluated also in the removal of MB under the same conditions. However, the pristine matrix was tested at first. PVA can adsorb MB thanks to its surface functional groups that could form strong hydrogen bonding with the dye structure^73^. The results displayed the maximum removal was ~ 30% after 20 min., Fig. 8[C, E]. Inclusion of only 4 *wt*% of ETB in the PVA matrix improved the rate of adsorption and led to eliminating all the dye molecules attributed to the synergistic effect, Fig. 8[D-E], i.e. the dye absorbance was not recorded over 40 min. Nonetheless, both films exhibited desorption behaviour by passing the reaction time. Table 2 presents a comparison between some different PVA composites-based adsorbents and the suggested one in terms of the removal efficiency against MB. As depicted, the prepared PVA nanocomposite in this study enjoys the highest removal %.

**Table 2:** Comparison of MB removal % of different studies.

Adsorbents	Conditions			Removal %	Reference
	Dye concentration (mg/L)	Adsorbent weight (g)	Treatment time (min.)		
PVA@WNS composite	5	0.25	120	82	^74^
PVA/CMC/TUR film	10	0.04	170	83	75
PVA/bagasse	181	1.0	70	92.8	^76^
Cellulose/PVA/ Graphite3D Porous Foam	150	0.06	300	96.2	^77^
PVA swollen gel	5	1	120	70	^78^
PVA/Graphene Oxide nanocomposite	80	1.5	2900	90	^79^
Mesoporous TiO_2_/ PVA nanocomposite	55	0.075	8.0	97	^80^
PVA/TiO_2_/microfibrillated cellulose	5	Thin Film	15	90	^81^
PVA/alginate/bentonite Hydrogel	50	1.5	300	95	^82^
PVA/TiO_2_nanowires/Biochar nanorods	20	0.5	20	100	This study

### Kinetics and isotherm

**Fig. 9:**
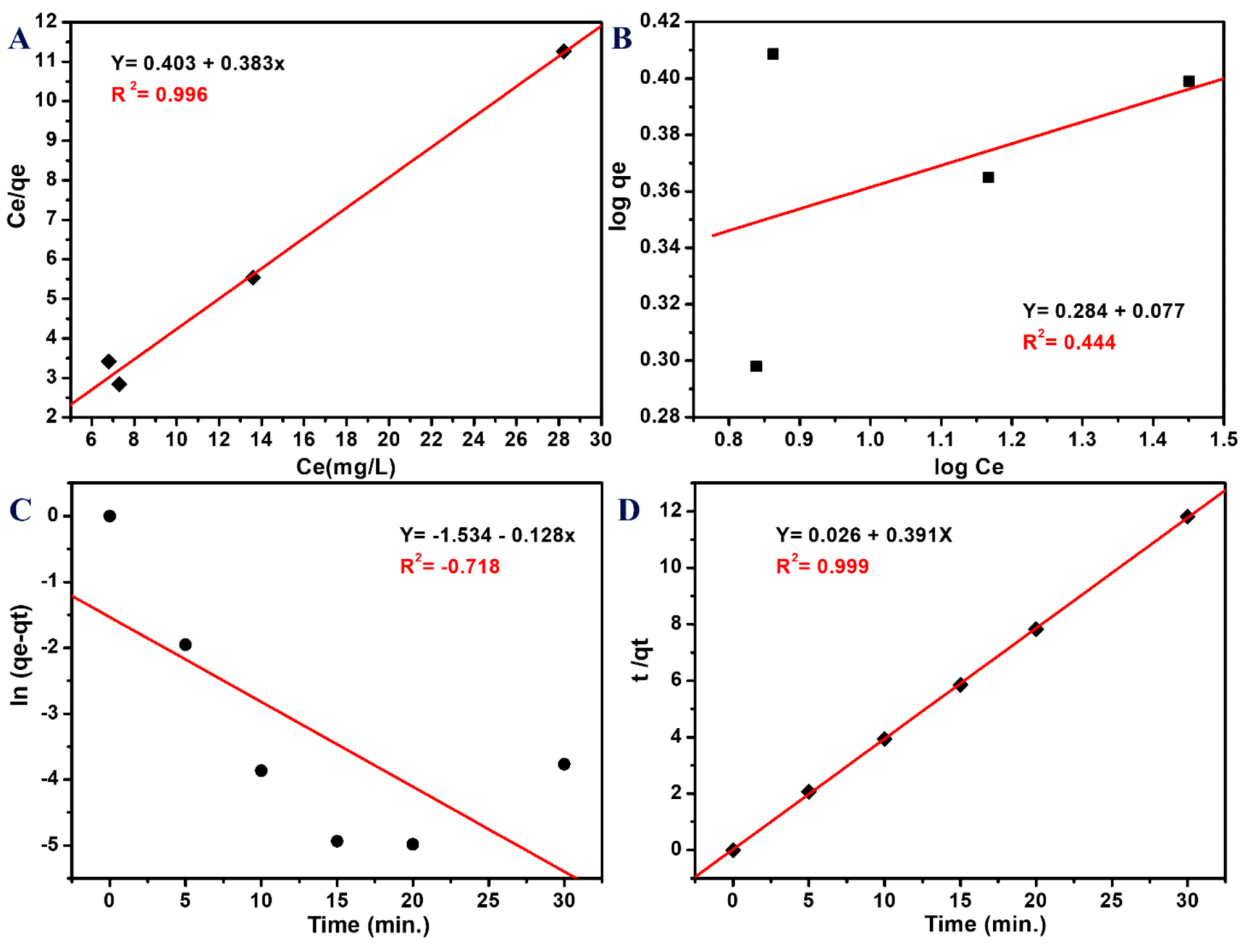
[**A**] Langmuir model, [**B**] Freundlich model, [**C**] Pseudo first-order, and [**D**] Pseudo second-order for the adsorption of MB over PVA nanocomposite film (EBT@PVA).

Adsorption isotherm is a most powerful way to understand how the pollutant molecules interact with the adsorbent surface. In this study, Langmuir and Freundlich models were considered for the MB-dye adsorption. Moreover, pseudo-first and pseudo-second order were used to analyze the adsorption kinetics. As shown in Fig. 9, the results suggested that the adsorption of methylene blue on the PVA nanocomposite film obeys Langmuir, monolayer adsorption, and follows the second-order kinetics as the values of the correlation coefficient were 0.996 and 0.999, respectively.

### Antimicrobial activity

**Fig. 10:**
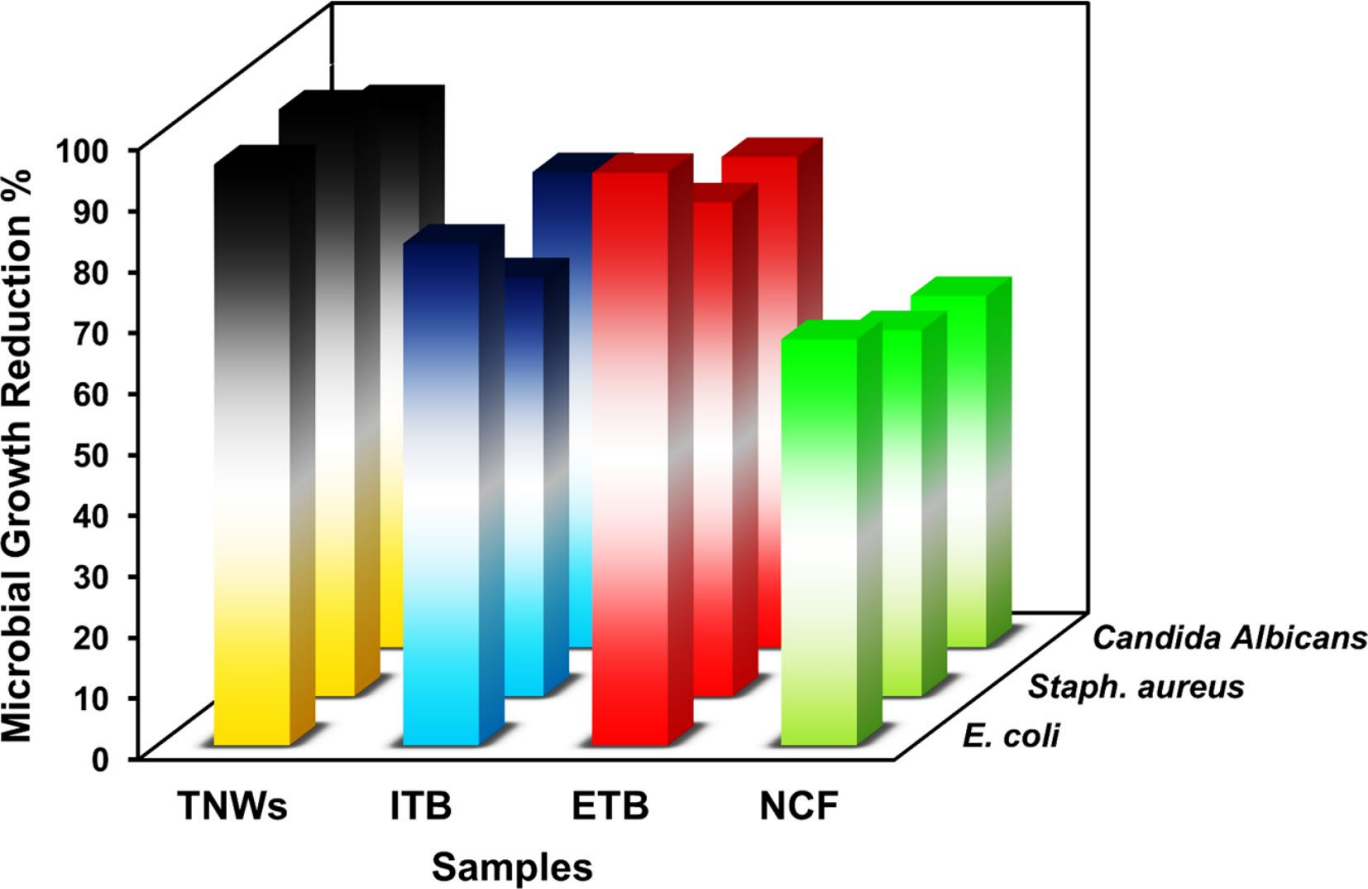
Antimicrobial activity for titanium dioxide nanowires (TNWs), in-situ modified titanium dioxide nanowires (ITB), ex-situ modified titanium dioxide nanowires (ETB), and nanocomposite PVA film (NCF) containing ETB.

The biocidal potential of titanate nanowires and their modified forms were investigated in Fig. 10 against three microorganisms i.e. *S. aureus* as Gram-positive bacteria, *E. coli* as Gram-negative bacteria, and *Candida Albicans* as a yeast. The results showed the highest activity against all tested strains. As widely reported, titanium dioxide has strong activity against pathogenic microbes. In a former study, TNWs were examined against the same organisms using the inhibition zone method as a powder sample^28^. However, in the present study, the investigation was performed utilizing the shake flask method followed by measuring the optical density. Indeed, the obtained results are harmonious with the reported findings. The mode of action for titanium dioxide as a promising antimicrobial agent was intensively investigated in the literature^83–85^. In brief, TiO_2_ could form reactive oxygen species (ROS) in the medium utilizing hydroxyl surface groups (cf. FTIR section). The produced ROS could react with the microbial cell membrane and penetrate the cell causing DNA destruction. Another reported mechanism was assumed by Cao and his coworkers^86^. They claimed that the electron clouds located at the surface of the cell membranes of the microbes can be transferred from the bacterial membrane to the titanium dioxide surface because of Schottky barrier effect since TiO_2_ is a semiconductor that has an electric potential, and the cell membrane can be considered as electron donors. The charge transfer process leads to create holes of positive charge on the cell surface. Also, this causes oxidative stress on the whole cell making the outer cell membrane more porous and eventually death of the cell by the leakage of cytoplasm^87^. On the other hand, modification of titanate nanowires with biochar nanorods either by in-situ or by ex-situ did not maintain the same activity. However, the ex-situ treatment showed better activity than in-situ one. This might be revealed to the consumption of surface functional groups of TiO_2_ in the interaction with biochar surface (cf. FTIR section) which might reduce the chances to form ROS and hence the activity decreased. However, the modified samples still exhibited strong biocidal performance since the decrease in activity was not comparable and cannot be considered. As reported, PVA has no action against microbes^88^. Therefore, people usually incorporate antimicrobial agent in the PVA matrix to impart it the property. In this study, ex-situ modified titanate nanowires were included in this matrix and the biocidal potential was studied. As shown in Fig. 10, the nanocomposite PVA film (NCF) showed good activity i.e. the microbial growth reduction reached 60%. It could be claimed that increasing filler loading % over 4 *wt*% would enhance its activity.

## Conclusions

In this study, titanium dioxide nanowires were prepared in sodium hydroxide solution under pressure using an autoclave at 240 °C for 72 h. Biochar nanorods were obtained via the combustion of rice husk at 400 °C under a nitrogen atmosphere. Then, titanate nanowires were modified with the nanorods of biochar by in-situ and ex-situ approaches. After that, the prepared titanate nanowires and their modifications were incorporated into the polyvinyl alcohol matrix to produce free-standing nanocomposite films. The microscopic images of TEM confirmed the successful preparation of titanium dioxide nanowires and biochar nanorods morphologies of average diameter ~ 45 and ~ 19 nm, respectively. SEM displayed a homogamous distribution of biochar nanorods between the titanate nanowires with the appearance of sodium silicate crystals formed during the in-situ modification step. FTIR showed strong hydrogen bonding and coordination bonds between the surface functional groups of both nanowires and nanorods. XRD exhibited shifting in the main peaks with a significant reduction in the crystallite size by ~ 28 and ~ 44% for in-situ modified and ex-situ modified nanocomposite, respectively. Also, the surface area and the total pore volume increased to a large extent. Thus, the synthesized nanocomposites showed a superior performance in the removal of methylene blue (20 ppm) after 20 min. treatment in the dark. The reaction order was pseudo-second order and follows the Langmuir model. The same activity was also observed for the PVA incorporated with 40 wt% of ex-situ modified nanocomposite. Against yeast along with Gram-positive and Gram-negative bacteria, the obtained nanocomposites showed strong antimicrobial activities close enough to that reported for the unmodified nanowires. Finally, from the study findings, it could be claimed that the ex-situ modified nanocomposite and its PVA film can be selected as the best choice to acte as a developed adsorbent with a high biocidal potential.

## Data Availability

All data generated or analyzed during this study are included in this article.
